# Computational characterization of Iron metabolism in the Tsetse disease vector, *Glossina morsitans*: IRE stem-loops

**DOI:** 10.1186/s12864-016-2932-7

**Published:** 2016-08-08

**Authors:** Zahra Jalali Sefid Dashti, Junaid Gamieldien, Alan Christoffels

**Affiliations:** South African Medical Research Council Bioinformatics Unit, The South African National Bioinformatics Institute (SANBI), University of the Western Cape, Robert Sobukwe Street, Bellville, South Africa

**Keywords:** *Glossina morsitans*, Hematophagy, Iron responsive elements, UTR, Gene regulation

## Abstract

**Background:**

Iron metabolism and regulation is an indispensable part of species survival, most importantly for blood feeding insects. Iron regulatory proteins are central regulators of iron homeostasis, whose binding to iron response element (IRE) stem-loop structures within the UTRs of genes regulate expression at the post-transcriptional level. Despite the extensive literature on the mechanism of iron regulation in human, less attention has been given to insect and more specifically the blood feeding insects, where research has mainly focused on the characterization of ferritin and transferrin. We thus, examined the mechanism of iron homeostasis through a genome-wide computational identification of IREs and other enriched motifs in the UTRs of *Glossina morsitans* with the view to identify new IRE-regulated genes.

**Results:**

We identified 150 genes, of which two are known to contain IREs, namely the ferritin heavy chain and the MRCK-alpha. The remainder of the identified genes is considered novel including 20 hypothetical proteins, for which an iron-regulatory mechanism of action was inferred. Forty-three genes were found with IRE-signatures of regulation in two or more insects, while 46 were only found to be IRE-regulated in two species. Notably 39 % of the identified genes exclusively shared IRE-signatures in other Glossina species, which are potentially Glossina-specific adaptive measures in addressing its unique reproductive biology and blood meal-induced iron overload. In line with previous findings, we found no evidence pertaining to an IRE regulation of Transferrin, which highlight the importance of ferritin heavy chain and the other proposed transporters in the tsetse fly. In the context of iron-sequestration, key players of tsetse immune defence against trypanosomes have been introduced namely 14 stress and immune response genes, while 28 cell-envelop, transport, and binding genes were assigned a putative role in iron trafficking. Additionally, we identified and annotated enriched motifs in the UTRs of the putative IRE-regulated genes to derive at a co-regulatory network that maintains iron homeostasis in tsetse flies. Three putative microRNA-binding sites namely Gy-box, Brd-box and K-box motifs were identified among the regulatory motifs, enriched in the UTRs of the putative IRE-regulated genes.

**Conclusion:**

Beyond our current view of iron metabolism in insects, with ferritin and transferrin as its key players, this study provides a comprehensive catalogue of genes with possible roles in the acquisition; transport and storage of iron hence iron homeostasis in the tsetse fly.

**Electronic supplementary material:**

The online version of this article (doi:10.1186/s12864-016-2932-7) contains supplementary material, which is available to authorized users.

## Background

Insect disease vectors are of immense significance to human health, with blood-feeding being a major habit exhibited by these insects. Blood feeding has evolved several times during the course of insect evolution and Dipterans constitute the most diverse and abundant order [1]. To understand the physiological adaptations of hematophagous insects in disease transmission, genome sequences of many disease-carrying blood feeding insects have been determined including *Anopheles gambiae*, *Aedes aegypti*, *Ixodes scapularis*, *Pediculus humanus* and *Glossina morsitans*. Furthermore, the insufficiency of current methods of disease control urges the need for devising new strategies. The genome sequences of insect disease vectors thus provide the ground for identifying novel targets. Furthermore, the genomic knowledge of these insects, when compared to their non-blood feeding counterparts, can provide compelling evidence about the physiological implications of blood feeding—specifically, the defense against host immune response, ingestion and digestion of the blood meal, and counteracting the deleterious effects of iron overload that accompanies such feeding habits. Among the hematophagous insects with their genome sequenced, tsetse flies pose important health problems and economic loss because of the transmission of Trypanosomes that affect both humans and livestock. Although the host blood meal is a rich source of iron required for tsetse’s developmental needs, excess iron can be detrimental [2]. Successful reproduction, symbiotic fitness and parasite survival entail a strict control of iron metabolism within the blood-feeding insects. Iron-metabolism is well-characterized in humans, however little is known in insects and more specifically blood-feeding insects. Human iron metabolism requires the function of several genes, including iron regulatory proteins, transferrin receptors (TfR), transferrin (Tf), ferritin, divalent metal transporter (DCT1), ferroportin, hephaestin, ceruloplasmin, iron-sulfur cluster proteins (ISC), frataxin, ferrochelatase, adenosine triphosphate (ATP)-binding cassette (ABC) and several others, the majority of which have no known homologs in insects. Based on the mammalian iron metabolism, iron uptake from transferrin involves the binding of Tf to the TfR. However, the lack of iron-delivery to insect organs through the receptor-mediated action of Tf poses the question about the possible conservation of the role of this gene in insects as transporters of iron. It may therefore be extrapolated that, in spite of, transporting iron, insect transferrin is not the predominant protein in performing iron transportation in these insects. In line with this, insect ferritin has been suggested as an alternative factor [3, 4]. Ferritin is a polymer involved in detoxification and iron storage. Despite the classic perception about the role of ferritin as mainly a cytoplasmic iron storage protein, insects have evolved such that ferritin is utilized as both an iron storage and an iron transporter [4]. Even though vertebrate’s ferritin mainly lacks signal peptides, insect’s ferritin including that of the tsetse fly contains secreted signals. Secreted insect ferritin has been found in high concentrations in the hemolymph, while cytoplasmic ferritin is significantly lower [5, 6].

Proteins involved in iron metabolism are regulated post-transcriptionally by the interaction of iron response elements (IREs) and Iron Regulatory Proteins (IRPs). IREs are RNA stem-loop structures, located in the untranslated regions (UTRs) of mRNAs involved in cellular iron homeostasis. This regulatory process takes place by the binding of IRPs to IREs in the 5’- and 3’-UTRs, which results in the expression initiation or suppression of the associated genes [7]. Some of the important structural features of IRE that allow for its recognition by IRP are, the presence of a terminal loop of sequences CAGUGH (H = U, C or A), a downstream stem of five base pairs that form an alpha-helix followed by a midstream C-bulge (C8) created by the conserved G-C bps, as well as sufficient base pairings before C8 to allow for IRE stability [8–10]. Additionally, the C14 and G18 nucleotides of the terminal loop, pair and form a pseudo-triloop (AGU) [8, 10–12]. Besides the canonical pattern defining IREs, several non-canonical forms have also been identified with an unpaired nucleotide buldge on the 3′strand of the upper stem or a mismatch pair in the upper stem. Initial reviews addressing the topic of IRE regulation were focused on two IRE-regulated genes, ferritin and TfR, known at the time [13]. Since then many mRNAs have been identified to be IRE-regulated, while many more remain to be characterized. Most of the known IREs to date have initially been identified in mammalian mRNAs.

The first functional IRE for insects, on the other hand, was identified in the 5'-UTR of Drosophila’s SDHB mRNA [14], which is not known to be IRE-regulated in humans and other mammals. It is thus apparent that in spite of its importance in survival, our knowledge of iron metabolism in insects is still obscure, while the exact mechanism of action and factors at play in determining the fate of blood meal in hematophagous insects remain elusive. Hence, identifying genes specific to the iron regulatory and metabolism pathways could reveal unique aspects of insect biology and provide clues to guide future research, that could lead to innovative control mechanisms. The focus of this work is to interrogate the UTR regions of the newly annotated *Glossina morsitans* genes for IRE signals to derive at signatures of regulation. This is achieved through the identification of IRE stem-loop structures and further characterization of other motif elements co-existing and co-regulating the putative iron metabolism genes.

## Results and discussion

### IRE-regulated genes in tsetse fly

The pattern matching analysis using canonical and non-canonical patterns associated with IREs reduced the total collection of *Glossina morsitans* UTRs to 6616. These were further assessed for the presence of IRE-stem loop structures using the SIRE (Searching for IREs) tool. Based on the score filtering, 150 putative IRE-containing genes were retrieved including 73 genes with 5′-IREs and 77 genes with 3′-IREs with high (See Additional file 1: Table S1) and medium scores containing a single mismatch (See Additional file 1: Table S2). IREs identified in the 5′UTRs comprise 17 canonical and 56 non-canonical structures, while IREs in the 3′UTRs include 20 canonical and 57 non-canonical patterns (See Additional file 2: Figure S1). To assess the accuracy of our predictions we examined the results using 1300 validated TSS positions [15]. Accordingly, 20 putative IRE-regulated genes have verified TSS with 12 having high score IREs, and 8, with Medium score IREs. Considering our choice of 1000 base pairs up- and down-stream of genes, the identified TSS (Transcription Start Site) for the IRE-regulated genes were approximately within range for 9 genes, while estimately 200 base pairs for the remaining genes (i.e., 11 genes). According to our results, the predicted IRE stem-loop structures are within the boundaries of the verified TSS positions except for 5, all of which were found to have a medium scored IRE in their UTRs. We can thus have confidence in the sensitivity of the high-scored predicted IREs while the validity of the medium-scored IREs should be further verified.

From the 150 putative IRE-regulated genes, two are known to be IRE-regulated, namely, ferritin heavy chain and myotonic dystrophy kinase-related CDC42-binding kinase alpha (MRCK-alpha), while the rest are novel (not previously published) with respect to their IRE-mechanism of regulation. Though literature suggests the linkage of some of these genes to mechanisms responsive to iron [16], no exclusive information on their post transcriptional IRE-regulation exists. Previous work by Ribeiro [16], assessing the expression of Anopheles transcripts in response to blood meal, supports the putative role of some of the IRE-regulated genes identified in this study, including the cuticular protein, myosin, importin, ATP-dependent RNA helicase, arrestin, acetyl-cholinesterase, ubiquitin-conjugating enzyme, cytochrome P450, acetyl-phosphatase, 40S ribosomal protein, 60S ribosomal protein, elongation factor 1 alpha, translation initiation factor, as well as several proteins of unknown functions. The blood meal-induced expression of these genes and their putative IRE mechanism of regulation proposed here, highlights their possible implication in controlling the toxic amount of blood meal iron ingested by the insect, hence its survival.

To gain insight into their role, the putative IRE-regulated genes were assigned to functional categories—such as functions in biosynthesis, cell envelop, metabolism, purines and pyramidines, transcription and translation, and transport and binding (See Additional file 2: Figure S2). Transcription, translation as well as metabolism were the over-represented functions with the *P*-values of 5.82e-5 and 1.39e-3 respectively. The biosynthesis and metabolism categories include components of amino acid biosynthesis, and biosynthesis of co-factors, as well as central intermediary metabolism, energy metabolism, and fatty acid metabolism. Furthermore, our results indicate that 58.94 % of the putative IRE-regulated genes are enzymes, which were further classified into ligase (25.84 %), lyase (16.85 %) and isomerase (11.23 %).

Using the sub-cellular localization analysis, we report that most IRE-regulated genes reside in the nucleus (26.49 %) and the cytosol (25.82 %), while others are distributed between endoplasmic reticulum, cytoskeleton, extracellular, mitochondria and plasma membrane (See Additional file 2: Figure S3). Furthermore, our data indicates that 17.21 % of the identified IRE-regulated genes are secreted proteins, of which 69.23 % are signal peptides and 30.76 % are signal anchors (Table 1). Among the predicted secretary proteins, the majorities are localized in the extracellular matrix. Furthermore, 61.53 % of the putative secretary proteins are cell envelope.

**Table 1:** Putative IRE-regulated secreted proteins identified in *Glossina morsitans*

Gene Name	Gene ID	GO	Localization
Shaking B St7-like protein Cuticle protein Ig-like domain containing protein Beta-Hexosaminidase-fdI Beta-Caroteine dioxygenase Gly-rich protein Insulin-like peptide 1 Integrator 4 Unknown Ferritin heavy chain Disulfide isomerase Glucosidase Cuticular protein 67B Angiotensin converting enzyme Retinoid and fatty acid binding glycoprotein Tetraspanin 42 Neuronal pentraxin 1 Glucose methanol choline oxidoreductase GH20979 Hmg-coenzyme A-isoform A Tm2d1 Unknown Lyzosyme NMDA receptor glutamate binding protein Gustatory receptor isoform C	GMOY004085 GMOY001551 GMOY000406 GMOY001317 GMOY001475 GMOY002975 GMOY003206 GMOY003945 GMOY005431 GMOY005797 GMOY008502 GMOY009591 GMOY006809 GMOY002936 GMOY000853 GMOY005442 GMOY003645 GMOY003829 GMOY000812 GMOY002247 GMOY005285 GMOY009232 GMOY003166 GMOY000103 GMOY004163 GMOY006209	Amino acid Biosynthesis Biosynthesis of cofactors Cell envelop Cell envelop Cell envelop Cell envelop Cell envelop Cell envelop Cell envelop Cell envelop Cell envelop Cell envelop Cell envelop Cell envelop Cell envelop Cell envelop Cell envelop Cell envelop Central intermediary metabolism Central intermediary metabolism Energy metabolism Energy metabolism Energy metabolism Transport and binding Transport and binding Transport and binding	Mitochondria Extracellular Extracellular Extracellular Extracellular Extracellular Extracellular Extracellular Extracellular Extracellular Extracellular Extracellular Cytosol Mitochondria E.R. E.R. Plasma membrane Plasma membrane Cytosol Cytosol Extracellular Extracellular Mitochondria Extracellular Plasma membrane Plasma membrane

Secreted proteins play critical roles in many biological processes such as cellular immunity and communication, hence contribute to the discovery of novel biomarkers [17, 18]. Thus, the identified IRE-regulated secreted proteins represent good candidates for future research on the development of control strategies, targeting iron-regulatory pathways in the tsetse fly.

### Tsetse’s mechanism of iron sequestration and trafficking

Proliferation is an important aspect of infection, in which the role of iron is well established [5]. The acquisition of iron is essential for the metabolic processes of the pathogen that have allowed them to adopt mechanisms to acquire protein-bound iron. To combat pathogen survival and invasion, iron sequestration is an important part of an innate immune response that is elegantly employed by insect vectors [19]. This may possibly be achieved through the function of several iron-binding proteins such as those identified in this study (Table 2). These include beaten pathIIa, sticks and stones, Ig-like domain containing protein, beta-carotene dioxygenase, MRCK-alpha, reverse transcriptase and defective proboscis extension response. The majority of these genes belong to the immunoglobulin superfamily with definitive roles in immune response.

**Table 2:** IRE-regulated immune response and stress response genes in *Glossina morsitans*

Gene Name	Gene ID	GO Category	Localization
Beaten path Sticks and stones Unknown IG-like domain containing protein Beta carotene dioxygenase Unknown Serine-threonine protein kinase Reverse transcriptase Defective proboscis extension response Ferritin heavy chain Glucose-fructose oxidoreductase Angiotensin-converting enzyme GH20979 Ring-box protein 2	GMOY005150 GMOY010203 GMOY003166 GMOY001317 GMOY002975 GMOY005797 GMOY001347 GMOY001995 GMOY005248 GMOY008502 GMOY011367 GMOY000853 GMOY002247 GMOY004737	Immune response Immune response Immune response Immune response Immune response Immune response Immune response Immune response Immune response Stress response Stress response Stress response Stress response Stress response	Plasma membrane Plasma membrane Mitochondria Extracellular Extracellular Extracellular Cytoplasm, nucleus Cytoplasm, nucleus Cytoplasm, nucleus Extracellular Extracellular E.R. Cytoplasm Cytoplasm

Hence, our findings, pertaining to the IRE-regulated immune response genes further points to the importance of these genes at the host-pathogen interface through the possible sequestration of iron from the invading pathogen. We also identified several stress-response genes under IRE-mechanism of regulation including the ferritin heavy chain, glucose-fructose oxidoreductase, angiotensin-converting enzyme, GH20979 and ring box protein2 (Table 2).

Considering the implications of iron overload in oxidative stress and the subsequent irreparable cellular damage [20], identifying IRE-regulated stress-response genes is expected. These genes could indeed play a role in iron-withholding response to deprive the invading pathogens of iron, hence protecting the insect vector. Though IRE regulation of ferritin heavy chain is well established [21] no knowledge of such regulation exists on the other stress response genes identified in this study, which represent novel targets for further investigation.

Genes responsible for the binding and trafficking of iron are vital for the survival of all organisms—especially, the insect vectors that are faced with the over-abundance of iron in their blood meal and the subsequent oxidative stress that may follow. Our work identify several cell envelope, transport, and binding proteins—the majority of which are localized in the extracellular environment and the plasma membrane respectively (Table 3). Besides their importance in protecting the vulnerable insect from the toxic effects of iron overload, we postulate that the identified genes may also function as antimicrobial peptides through withholding ferric ion from the invading pathogen i.e., trypanosomes. In line with this, the work of Lehane [22] on the expression analysis of putative immune response genes in the midgut of tsetse fly further supports the role of IRE in regulating ATP synthase, and 60S ribosomal proteins. Lehane and colleagues [22] showed that the self-cured flies induce an oxidative stress response following trypanosome infection [22]. The genes cited above may function at the host-pathogen interface to fight infection either through iron sequestration or the production of reactive oxygen species (ROS) and imperative targets for future studies.

**Table 3:** Putative genes involved in iron trafficking in *Glossina morsitans*

Gene Name	Gene ID	GO category	Localization
Glucosidase Cytochrome-P450 Angiotensin-converting enzyme Retinoid & fatty acid glycoprotein Cuticle protein Ig-like domain containing protein Beta hexosaminidase fdI Beta carotene dioxygenase Gly-rich protein Insulin-like peptide1 Integrator 4 Unknown Ferritin heavy chain Disulfide isomerase GJ22290 Glucose fructose oxidoreductase Cuticular protein 67B GI16026 Tetraspanin42 Neuronal-pentraxin1 Lyzosyme Cytochrome B561 Unknown Hypothetical protein NMDA-receptor glutamate binding Gustatory receptor isoform C Serine/threonine protein kinase Hydroxy-tryptamine-receptor Unc-50	GMOY006809 GMOY010179 GMOY000853 GMOY005442 GMOY000406 GMOY001317 GMOY001475 GMOY002975 GMOY003206 GMOY003945 GMOY005431 GMOY005797 GMOY008502 GMOY009591 GMOY007668 GMOY011367 GMOY002936 GMOY004434 GMOY003645 GMOY003829 GMOY000103 GMOY010183 GMOY003757 GMOY006990 GMOY004163 GMOY006209 GMOY001347 GMOY004778 GMOY011894	Cell envelop Cell envelop Cell envelop Cell envelop Cell envelop Cell envelop Cell envelop Cell envelop Cell envelop Cell envelop Cell envelop Cell envelop Cell envelop Cell envelop Cell envelop Cell envelop Cell envelop Cell envelop Cell envelop Cell envelop Transport and binding Transport and binding Transport and binding Transport and binding Transport and binding Transport and binding Transport and binding Transport and binding Transport and binding	Cytosol Cytosol/Nuclear E.R. E.R. Extracellular Extracellular Extracellular Extracellular Extracellular Extracellular Extracellular Extracellular Extracellular Extracellular Extracellular Extracellular Mitochondria Mitochondria Plasma membrane Plasma membrane Extracellular Extracellular Nuclear Nuclear Plasma membrane Plasma membrane Plasma membrane Plasma membrane Plasma membrane

One of the well-studied genes presented in our results namely ferritin heavy chain is involved in iron storage and transport, was found not only to be confined to the cytosol but also to be present in the extracellular environments. Additionally, to confirm and complement the previous finding, the work presented here has recognized the implication of ferritin heavy chain in secretary pathways. As previously described by Nichol [4], this observation, though common to several insects, is in contrast to the vertebrates ferritin heavy chain, which is mainly cytosolic. Furthermore, despite the evident role of Tf in iron transport among various species, lack of evidence on identifying an IRE stem-loop in the UTRs of Glossina Tf in this study may point to the importance of ferritin heavy chain and the other proposed transporters in the tsetse fly. This could further imply Tf-regulation by mechanisms other than IREs. Additionally, the absence of Tf-receptor in the genome of Glossina, which is evolutionarily, in line with the loss of the C-terminal lobe from insect-Tf’s [2], further highlights the significance of the identified genes as alternative mechanisms by which iron regulation is maintained.

We further identified IRE stem-loop structures in the UTRs of several energy metabolism and mitochondrial genes (Table 4), which supports the role of iron in modulating energy metabolism of ATP formation via oxidative phosphorylation [23].

**Table 4:** IRE-regulated energy metabolism genes in *Glossina morsitans*

Gene Name	Gene ID	Localization
Hypothetical protein	GMOY010181	Plasma membrane
LIX1-like protein	GMOY003300	Nucleus
Bruno-3 transcript	GMOY007718	Nucleus
20s proteasome regulatory subunit beta	GMOY004532	Mitochondria
Hmg-coenzyme A-isoform A	GMOY005285	Extracellular
Folypolyglutamate synthase	GMOY005208	Cytosol
Serine-pyruvate mitochondrial	GMOY008920	Cytosol
Reverse transcriptase	GMOY001995	Cytosol
Phenylalanyl-tRNA synthase subunit beta	GMOY004566	Extracellular
Tmd2d1	GMOY009232	Extracellular
Unknown	GMOY003166	Mitochondria
Serine/threonine protein kinase	GMOY001347	Cytosol, Nucleus
Coiled-coiled helix	GMOY011710	Nucleus
Gj11024	GMOY010160	Mitochondria
Heterochromatin-associated protein	GMOY003696	Cytosol, Nucleus
Hypothetical protein	GMOY000545	Cytosol
Acetyle Cholinesterase	GMOY002161	Cytosol
Synaptotagmin	GMOY007406	Cytosol

### Co-regulators of IRE-regulated genes

To further characterize the post-transcriptional regulatory elements governing iron metabolism in *Glossina morsitans*, the UTRs of IRE-regulated mRNAs, or putative iron metabolism genes were analyzed for the presence of other enriched regulatory motifs. Multiple EM in Motif Elicitation (MEME) suite [24], was used for de novo motif discovery, as well as the UTRScan to search for the presence of experimentally validated elements. Seven sequence motifs in the 5′- and 3′-UTRs of Glossina genes were identified using MEME, while twelve were found using UTRScan (See Additional file 3: Table S3, S4). Besides elements commonly present in the UTRs of most mRNAs such as uORF and PAS located in the 5′- and 3′- UTRs, respectively, we identified other enriched elements including IRES, crcB and Bacteroid-trp-like RNA motif present in the 5′UTRs; as well as FIE3, GAIT, GY-box, Brd-box, K-box, ARE, and CPE in the 3′UTRs. Furthermore, UNR-bs and SXL-bs were commonly identified in both UTRs. We, therefore, propose the over-representation of immune response regulatory elements such as GAIT and ARE as well as reproduction regulatory elements including FIE3, CPE, and SXL-bs. Additionally, we identified three motifs in the 3′UTR of IRE-regulated genes that are complementary to the 5′-end of miRNAs, namely GY-box, Brd-box, and K-box motifs. These elements have previously been identified in Drosophila, mosquitoes, bees, moth and several other insect species [25]. However, no evidence on their interplay with IRE-mechanism of regulation exists to date. These motifs are often found in the UTR of Notch target genes that allow for sufficient miRNA-mediated regulation [25–28]. The involvement of Notch signalling in various biological processes has been well established, including embryogenesis, development of the central nervous system and function, cardiovascular and endocrine development [29–31].

### IRE-regulated Hypothetical proteins

As part of the 150 genes identified, several hypothetical/uncharacterized proteins were classified as IRE-regulated (Table 5), hence many have a putative role in tsetse’s iron metabolism. Some of these genes have no known orthologs, and therefore, are unique to Glossina, while others are conserved hypothetical proteins. We assessed the domain architecture of these genes and their associated orthologs are presented in Table 5. Furthermore, to understand the putative role of these hypothetical/uncharacterized proteins we examined the regulatory elements identified in the UTR regions of these genes (Table 5). Besides their possible IRE mechanism of regulation, 9 hypothetical/uncharacterized proteins were identified with UNR-bs and SXL-bs elements in their 5′- and 3′-UTRs. In human, UNR-bs is involved in c-Fos protein destabilization as well as translation repression of the poly(A)-binding protein (PABP) [32, 33]. In vivo and in vitro studies have identified UNR as a critical factor in major coding-region determinant of instability (mCRD)-mediated mRNA turnover due to its function as an mCRD-binding protein as well as a PABP -interacting protein. As a result, mCRD/UNR complex is considered as the responsible unit in the formation of deadenylation/decay mRNP complex [32].

**Table 5:** IRE-regulated hypothetical proteins in *Glossina morsitans*

Gene ID	Gene Ontologies	InterPro Domains	Orthologs	Regulatory Motifs	
				5′UTR	3′UTR
GMOY005042	-	IPR009533: Protein of Unknown Function DUF1151	Yes	-	PAS, SXL_BS
GMOY006990	-	-	Yes	UNRbs, SXL_BS, uORF	-
GMOY000545	-	-	Yes	Bacteroidtrp, UNRbs, uORF	-
GMOY000910	-	-	No	uORF	-
GMOY001601	-	IPR022096: Myotubularin protein; IPR005578: Hrf1	Yes	Bacteroidtrp, UNRbs, uORF	-
GMOY002533	-	-	Yes	-	PAS
GMOY006141	GO:0006278: RNA-dependent DNA replication	IPR000477: Reverse transcriptase; IPR016193: Cytidine deaminase-like; IPR012337: Ribonuclease H-like domain; IPR025398: Domain of unknown function DUF4371; IPR008906: HAT dimerisation; IPR001888: Transposase, type 1	Yes	Bacteroidtrp, IRES, uORF	-
GMOY010181	-	-	No	PAS	-
GMOY003166	-	-	No	IRES, uORF	-
GMOY003757	-	-	No	PAS, UNRbs	-
GMOY005797	-	-	No	uORF	-
GMOY007187	-	-	Yes	uORF	-
GMOY011151	MF: GO:0016874: ligase activity; BP: GO:0022008: neurogenesis	IPR016177: DNA-binding, integrase-type; IPR025884: Methyl-CpG binding protein 2/3, C-terminal domain; IPR001739: Methyl-CpG DNA binding	Yes	-	PAS
GMOY006965	-	-	Yes	UNRbs, IRES, SXL_BS, uORF	-
GMOY008376	-	-	Yes	UNRbs, IRES, uORF	-
GMOY008535	GO:0007165: signal transduction	IPR011022: Arrestin C-terminal-like domain; IPR011021: Arrestin-like, N-terminal; IPR014756: Immunoglobulin E-set; IPR006612: Zinc finger, C2CH-type	Yes	-	-
GMOY002247	MF: GO:0004930: G-protein coupled receptor activity, BP: GO:0008355: olfactory learning	IPR019326: Protein of unknown function DUF2369; IPR003961: Fibronectin, type III; IPR000276: G protein-coupled receptor, rhodopsin-like; IPR017452: GPCR, rhodopsin-like, 7TM	Yes	-	CPE, PAS, SXL_BS
GMOY004434	-	-	Yes	uORF	-
GMOY011791	-	IPR022110: Casc1 domain	Yes	-	-
GMOY007668	GO:0016491: oxidoreductase activity	IPR002018: Carboxylesterase, type B	Yes	Bacteroidtrp, UNRbs, uORF	-

Additionally, in Drosophila the translational repression of male-specific-lethal 2 (MSL2) mRNA by Sex-lethal (SXL) requires the functioning of UNR [34]. MSL2 is a component of Drosophila dosage compensation complex that regulates the expression of X-linked genes between males (XY) and females (XX). This is achieved through promoting hyper-transcription of the single male X chromosome [35]. Furthermore, the SXL binding site (SXL-bs) where SXL binds have been suggested to suppress translation [36–38]. The binding of SLX to 3′-UTR inhibits the 43S ribosomal complex recruitment to the mRNA, while it’s binding to the 5′-UTR prevents the scanning of complexes that have escaped the 3′-mediated inhibition [39, 40]. The presence of UNR-bs and SXL-bs in the UTRs of IRE-regulated hypothetical/uncharacterized genes further point to the putative role of these genes and the implications of iron in the reproductive aspects of insect biology.

Additionally, we identified 4 hypothetical/uncharacterized proteins with IRES element in their 5′UTRs. These include GMOY006141 (with Reverse transcriptase domain), GMOY003166, GMOY006965, and GMOY008376. IRES is an RNA element that allows internal ribosomal recruitment and translation initiation in the middle of a messenger RNA (mRNA) and is used as a mechanism to increase translation of certain proteins [41, 42]. Though, the mechanism of action for these genes are not evident the presence of both IRE and IRES elements in the UTR of these genes highlight their putative role in blood meal-induced set of events that may control and prevent iron toxicity.

### Evidence of a putative IRE-mechanism of regulation in orthologs of Glossina morsitans iron metabolism genes

With current literature devoid of evidence supporting the association of a number of putative IRE-regulated genes identified in this study to mechanisms relating to iron homeostasis, we further evaluated our findings through assessing their homologs in other insect species. Accordingly, genes with orthologs in *D. melanogaster* and *M. domestica* as non-blood feeding insects, as well as all the blood feeding insect species with genomic data available in Vectorbase (https://www.vectorbase.org/genomes), were assessed. The main drawbacks in this analysis were the varying number of genes with orthologs in different species, as well as lack of an annotated UTR sequence for some of the queried putative IRE-regulated genes in the species under study. Accordingly, from the 150 putative IRE-regulated genes in *Glossina morsitans*, 43 were found with IRE-signatures of regulation in 2 or more insects (Table 6), while 46 were only found to be IRE-regulated in two species (Table 7). Notably 39 % of the identified genes exclusively shared IRE-signatures in other Glossina species, which are potentially Glossina-specific adaptive measures in addressing its unique reproductive biology and blood meal-induced iron overload. Though their possible IRE-regulated mechanism of action cannot be disregarded, the remaining 61 genes without a co-occurrence support from other insect species should be treated as ambiguous, in the absence of additional supporting evidence.

**Table 6:** IRE-regulated genes supported by more than one ortholog with putative IRE-mechanism of regulation

*G. morsitans* ID	Orthologs	Gene name
GMOY000584	GAUT041244, GBRI044910, FBgn0030479, GFUI001750, MDOA005113, GPAI044041, GPPI009094	Uncharacterized protein
GMOY000853	GFUI033358, GPAI038270, GPPI044829	Angiotensin-converting enzyme-like
GMOY001045	GAUT008477, FBgn0033972, GPAI030524	Serine/threonine-protein kinase
GMOY001046	GAUT008476, GFUI050919, GPAI030522	GK22170
GMOY001347	GAUT016020, GPPI003550	GK25296
GMOY001475	GAUT026625, MDOA012345, GPPI050112	Oligosaccharidolytic beta-N-acetylglucosaminidase-like
GMOY001551	GAUT042169, FBgn0037026, GPAI032477	ST7 homolog (LOC101894805), mRNA
GMOY001601	GAUT035263, GFUI018313, GPAI012880, GPPI008027	Uncharacterized protein
GMOY002533	GBRI014000, GFUI050716	PFC0760c-like (LOC101898723)
GMOY003206	GFUI043226, GPPI017270	Glycine-rich cell wall structural protein 1-like (LOC101892314)
GMOY003449	GAUT016397, GFUI025828, GPAI018591	Autophagy protein 5-like (LOC101450692)
GMOY003491	GAUT035878, FBgn0267429, GFUI053351, GPPI016882	Proton-associated sugar transporter a-like
GMOY004282	GAUT042374, FBgn0015396, GPAI003358	Fork head box transcription factor
GMOY004296	AGAP007391, GAUT050548, FBgn0033086	GK23085 (Dwil\GK23085)
GMOY004905	GBRI004313, FBgn0037298	Saccharopine dehydrogenase domain containing protein
GMOY005336	GAUT008867, GPAI006027	Mitochondrial-processing peptidase subunit beta-like
GMOY005442	GAUT005852, GBRI028066, FBgn0087002, GPAI017174	Retinoid-and fatty acid-binding glycoprotein
GMOY005513	GAUT045304, GPAI000999	26 s proteasome regulatory complex subunit psmd5
GMOY005545	GAUT040539, GPAI013102	Abdominal-B protein
GMOY006327	FBgn0039705, GFUI011317, GPPI037740	Autophagy-related protein
GMOY006724	FBgn0015380, GFUI015685, GPPI027704	Receptor tyrosine kinase
GMOY006808	GFUI015876, GPPI047634	Eukaryotic translation initiation factor 3
GMOY007858	GAUT044979, GPAI037485	Tetratricopeptide repeat protein
GMOY007975	GBRI005362, GPPI034851	Clock work isoformb
GMOY008151	GFUI047759, GPAI027699	General transcription factor IIH subunit1
GMOY008502	AGAP002465, GAUT049017, GBRI029736, FBgn0015222, GFUI020912, MDOA013394	Ferritin heavy chain-like protein
GMOY008535	GAUT022428, GFUI019745, MDOA001327, GPAI008100	Arrestin domain-containing protein 5-like
GMOY008920	GBRI012722, FBgn0014031	Serine-pyruvate mitochondrial
GMOY010282	GBRI044609, GFUI013632, MDOA015279	Amino-acylase-1
GMOY011648	FBgn0038861, GFUI002356, GPPI028265	trna(uridine-2-o-)-methyl transferase trm7
GMOY011720	AGAP004310, GBRI037168	PERQ amino acid-rich with GYF domain
GMOY000357	SCAU003080, SCAU008783	dynein lightchain
GMOY001186	AALB004468, AALF006549, SCAU012681	DNA replication licensing factor mcm7
GMOY003300	AAEL001809, AATE008804, ACOM040440, AMEM009039, AMIN001566	LIX1-like protein
GMOY005208	AAEL012882, AMIN007515	folylpolyglutamate synthase, mitochondrial like
GMOY006619	AATE010711, AFAF007359	stoned isoform e
GMOY006809	ASIS009945, ASTE001310	Glucosidase
GMOY007718	ACOM024486, AMIN011443	bruno-3 transcript
GMOY008670	ACOM039036, ACOM039038	n-methyl-d-aspartate receptor glutamate-binding subunit
GMOY009423	AALB003034, SCAU001454	E3 ubiquitin-protein ligase
GMOY009591	AAEL000641, AATE013365, AMIN008920	disulfide isomerase
GMOY010018	AALB006737, AATE019498, ASTE010551	phd finger protein
GMOY011894	AAEL013898, AATE018226, ACOM037504, AMEM017576	unc-50

The following species were identified to share IRE-signatures with Glossina morsitans genes, namely: D. melanogaster (FBgn), M. domestica (MDOA), A. gambiae (AGAP), A. atroparvus (AATE), A. albimanus (AALB), A. coluzzii (ACOM), A. merus (AMEM), A. minimus (AMIN), A. farauti (AFAF), A. sinensis (ASIS), A. stephensi (ASTE), A. aegypti (AAEL), A. albopictus (AALF) G. austeni (GAUT), G. brevipalpi (GBRI), G. fuscipes (GFUI), G. palidipes (GPAI), G. palpalis (GPPI), S. calcitrans (SCAU)

**Table 7:** IRE-regulated genes supported by a single ortholog with putative IRE-mechanism of regulation

*G. morsitans* ID	Orthologs	Gene Name
GMOY000103	GPAI010329	Lysozyme
GMOY000519	GAUT036378	Nuclear pore complex protein
GMOY001007	FBgn0034396	Kelch-like protein diablo
GMOY001576	FBgn0015278	Phosphoinositide3-kinase
GMOY002082	GPAI012017	Zinc finger protein2
GMOY002199	MDOA001720	Elongation factor1 alpha
GMOY002247	AGAP028539	GH20979
GMOY002470	GBRI013281	Importin9
GMOY002504	GFUI013783	DNA polymerase epsilon
GMOY002505	GFUI013765	ATP synthase subunit cf6
GMOY002713	GPPI004271	Spire
GMOY002753	GPPI009339	Serine-rich adhesin for platelets-like
GMOY003115	GAUT029025	Arp 23 complex subunit arpc5
GMOY003531	GAUT027886	ATP-dependent RNA helicase dhx 36-like
GMOY003645	GBRI002560	Tetraspanin42
GMOY003945	GPAI002313	Insulin-like peptide 1
GMOY004163	FBgn0260777	Nmda receptor glutamate-binding chain
GMOY004507	GPAI006744	Saccharopine dehydrogenase domain-containing protein
GMOY005027	FBgn0031888	Probable serine/threonine-protein kinase
GMOY005042	AGAP000675	Hypothetical conserved protein
GMOY005248	FBgn0040726	Defective proboscisextension response
GMOY005401	GAUT045141	Hypothetical conserved protein
GMOY005444	GPAI022424	GH23986
GMOY005485	FBgn0014127	Barren
GMOY005887	GBRI011291	Nucleosome remodeling factor - isoform a
GMOY006156	FBgn0034418	CG15118
GMOY006990	MDOA011437	Hypothetical conserved protein
GMOY006996	FBgn0036882	150 Kda dynein-associated polypeptide
GMOY007017	GFUI040511	Ubiquitin-conjugating enzyme9
GMOY008376	GAUT051004	GG21264
GMOY009258	GBRI017686	Brain chitinase and chia
GMOY010325	FBgn0052062	Ataxin-2 binding protein
GMOY010911	GPAI043869	Pentatricopeptide
GMOY011040	GPAI043262	UBX domain-containing protein 7
GMOY011151	AGAP003983	GF18386
GMOY011465	FBgn0038532	Zinc finger protein 106
GMOY000812	AALF015524	Glucose-methanol-choline oxidoreductase
GMOY002244	AATE008428	f-box protein
GMOY002975	AEPI008885	beta-carotene dioxygenase
GMOY004236	ASIS018672	acylphosphatase
GMOY004532	AATE002806	20s proteasome regulatory subunit beta type
GMOY004737	AALF022793	ring-box protein2
GMOY006264	ISCW017255	LIM domain protein
GMOY006321	AAEL001635	gamma-tubulin ring complex
GMOY007780	SCAU003198	rhomboid
GMOY011965	AAEL017516	60s ribosomal protein l23

The following species were identified to share IRE-signatures with Glossina morsitans genes, namely: D. melanogaster (FBgn), M. domestica (MDOA), A. gambiae (AGAP), A. atroparvus (AATE), A. albimanus (AALB), A. coluzzii (ACOM), A. merus (AMEM), A. minimus (AMIN), A. farauti (AFAF), A. sinensis (ASIS), A. stephensi (ASTE), A. aegypti (AAEL), A. albopictus (AALF) G. austeni (GAUT), G. brevipalpi (GBRI), G. fuscipes (GFUI), G. palidipes (GPAI), G. palpalis (GPPI), S. calcitrans (SCAU)

## Conclusions

Lack of conservation of the key players between human and insect iron metabolism indicates that iron regulation is conveyed by different mechanisms. As a co-factor in the kreb cycle and oxidative phosphorylation, iron is critical in the production of ATP for tsetse’s energy requirements. Furthermore, the implications of iron in processes such as the immune response, maintenance of circadian rhythms as well as developmental and aging-related processes are well established [43]. Such broad range of functional categories is also evident in our findings pertaining to the putative IRE-regulated genes in tsetse fly. The proposed participation of a number of these genes in secretory pathways provide a new insights into iron metabolism for Glossina, and possibly, other blood feeding insects.

Several lines of evidence exist in understanding the fate of blood meal iron in mosquitoes, however no such extensive work has been done in the tsetse fly. Research in understanding iron metabolism in insects has mostly focused on a few genes including ferritin [44–46], transferrin [47] [6], iron regulatory protein 1 [48], and the divalent metal transporter1 (DMT1) [49]. However, iron regulation in tsetse fly in the context of IRE-regulation has not been well addressed to date. In line with this, our findings provide a unique perspective on the role of iron in tsetse biology and further understanding of how it utilizes iron and direct it towards egg development, while preventing iron-induced toxicity. This broad knowledge base provides the ground for future research in the area of vector control. Furthermore, our results attest to the tight co-regulation that exists between iron metabolism and immune response as well as reproduction processes in the tsetse fly. In light of recent findings where iron has been shown to regulate the activity of the miRNA pathway [50], our results, further support the link between iron regulation and miRNA activity, through the identification of miRNA binding motifs in the UTRs of several IRE-regulated genes in Glossina.

Finally, the knowledge provided here conveys important clues to guide future research in better understanding key players in the acquisition, transport and storage of iron in *Glossina morsitans*, and their imperative role in orchestrating a network of events leading to iron metabolism and regulation.

## Methods

### IRE prediction

In the absence of a well annotated UTR dataset for 12220 Glossina genes, the 5′- and 3′-UTRs were defined as a 1000 bps up- and down-stream of a gene respectively. A perl script was written to retrieve the UTR sequences. The script scans through the GFF file for the word “CDS” and extracts a 1000 bps up- and down-stream using the genomic sequence. The UTR sequences are saved in a fasta-formatted file, which can be used for further analysis. The program “dna-pattern” as part of the RSAT tools [51] was used to scan UTR sequences with string-based patterns of IREs. These patterns were provided to RSAT as regular expressions using IUPAC-IUB symbols, covering a wide range of canonical, non-canonical and SELEX-based IRE patterns [52]. RSAT pattern matching analysis was used to refine the putative list of UTR sequences containing IRE-like patterns. The UTRs of Glossina morsitans were screened for the presence of IRE stem-loop structures using Search for IREs (SIRE) [52], whereby the 3′- and 5′-UTR sequences were separately provided as inputs. The predicted IREs are then folded using “RNAfold” as part of the Vienna RNA package [53], implemented in SIRE. The filtering step was carried out and IREs with “High” score were retrieved. High scoring IREs refer to those predicted to have a canonical form of IRE, and either none or a single mismatch or bulge. To account for genes with IRE-like elements and considering that, ferritin IRE was ranked “Medium” by SIRE as part of the filtering step, genes ranked as “Medium” with no mismatch were also considered for further analysis. Medium scoring IREs on the other hand refer to those structures that partially fulfil known IRE features, such as those identified through SELEX experiments (non-canonical forms). The identified IRE sequence patterns were collected in fasta format file for further analysis. Additionally, the protein sequences associated with the identified genes were extracted from the available Glossina peptide file for further analysis.

### Functional classification

To gain insight into the function of the putative IRE-regulated genes, ProtFun v.2.2 [54] was used to assigns genes to functional categories. Furthermore, SignalP-NN v.4.1 [55] and TMHMM v.2.0 [56] as implemented in ProtFun allowed for the inference of putative secreted proteins, and their further classification as signal peptides or signal anchors. We also carried out functional enrichment analysis using BiNGO [57] as implemented in Cytoscape.

### Predicting sub-cellular localization

To predict the sub-cellular localizations of the identified IRE-regulated genes WoLF-PSORT v.0.2 [58] was used. The fasta-formatted protein sequences were provided as input to the program and the results were captured in a localization feature table, while localizations with the highest probability were considered.

### Motif enrichment analysis

The UTR sequences of the predicted IRE-regulated genes were retrieved and further assessed for the enrichment of other regulatory motifs, whereby the background model was predicted using the fasta-get-markov method as implemented in the MEME suite [24]. Motif discovery was carried out using MEME v4.9.0, with the minimum and maximum motif width of six and thirty respectively. The p-value cut-off to consider a motif significant was specified as 10-4. Parameter specification was based on the visual inspection of the alignment. To annotate the identified motifs, a meme-readable database of UTR elements is required. As such, a database of all known UTR elements was created, through retrieving their sequences from UTRsite (http://utrsite.ba.itb.cnr.it/) and RFAM database (http://rfam.sanger.ac.uk/). The gathered sequences for each of the known elements were searched for an overrepresented pattern using MEME, by specifying the minimum and maximum width associated with each element. This was carried-out for 51 5′-UTR and 79 3′-UTR elements. The resultant matrix files were then summarized into a single meme file using meme2meme program. The annotation of the identified motifs was then carried out through their comparison to this database of known UTR elements, using the Find Individual Motif Occurrences (FIMO), as implemented in MEME suite. Accordingly, the best matching motif (based on the *q*-value) was used to annotate each of the identified motifs.

### Prediction of known UTR elements

To complement the results of MEME, the UTR sequences containing putative IRE stem-loop structures were further analyzed using UTRScan, which searches for the previously identified UTR patterns that are available in UTRsite [59]. The UTR sequences of Glossina genes, identified to be IRE-regulated were provided as input, while 5′- and 3′-UTR sequences were searched in separate runs to differentiate between patterns that are specific to each of these regions and/or those that are commonly found in both the 5′- and 3′-UTRs.

## Abbreviations

ABC, ATP-binding cassette; DCT1, divalent metal transporter; FIMO, find individual motif occurrences; IRE, iron response element; ISC, iron-sulfur cluster; MEME, multiple EM in motif elicitation; MSL2, male-specific-lethal 2; PABP, Poly(A)-binding protein; ROS, reactive oxygen species; SIRE, search for IREs; SXL, sex-lethal; Tf, transferrin; TfR, transferrin receptor; TSS, transcription start site; UTR, untranslated region

## Additional files

Additional file 1: Table S1. High-ranked IRE-regulated genes in *Glossina morsitans*. A summary of the identified High-ranked IRE-regulated genes in Glossina, including their gene names and IRE sequence patterns. **Table S2.** Medium-ranked IRE-regulated genes in *Glossina morsitans*. A summary table of the medium-ranked IRE-regulated genes, and their identified IRE sequence patterns. (DOCX 3336 kb) Additional file 2: Figure S1. Pattern distribution of the identified IRE stem loop structures. Bar graph presentation of the identified IRE-regulated genes, having canonical and non-canonical IREs. **Figure S2.** GO-category assignments of IRE-regulated genes in Glossina. A bar graph presenting the number of putative IRE-regulated genes associated with each GO category. **Figure S3.** Sub-cellular localization assignments of IRE-regulated genes in Glossina. A bar graph, presenting the number of putative IRE-regulated genes and their associated subcellular localizations. (DOCX 357 kb) Additional file 3: Table S3. Motifs identified in the 5′-UTRs of IRE-regulated genes in Glossina, using MEME and UTRScan. Table S3 presents a list of motifs identified in the 5′UTRs of putative IRE-regulated genes. **Table S4.** Motifs identified in the 3′-UTRs of IRE-regulated genes in Glossina, using MEME and UTRScan. A summary of the regulatory motifs identified in the 3′UTRs of IRE-regulated genes. (DOCX 2123 kb)
