# Supplementary material for: Computational characterization of Iron metabolism in the Tsetse disease vector, Glossina morsitans: IRE stem-loops
Source: BMC Genomics. 2016 Aug 8;17:561. doi: 10.1186/s12864-016-2932-7 (PMC4977773; doi:10.1186/s12864-016-2932-7)
Supplement: Additional file 1: Table S1. — High-ranked IRE-regulated genes in Glossina morsitans. A summary of the identified High-ranked IRE-regulated genes in Glossina, including their gene names and IRE sequence patterns. Table S2. Medium-ranked IRE-regulated genes in Glossina morsitans. A summary table of the medium-ranked IRE-regulated genes, and their identified IRE sequence patterns. (DOCX 3336 kb) [file 12864_2016_2932_MOESM1_ESM.docx]

**Table. S1**: High-ranked IRE-regulated genes in *Glossina morsitans*. High scoring IREs refer to those predicted to have a canonical form of IRE, and either none or a single mismatch or bulge.

**Table. S2**: Medium-ranked IRE-regulated genes in *Glossina morsitans*. Medium scoring IREs refer to stem loops that partially fulfill known IRE features, such as those identified through SELEX experiments (non-canonical forms).
