# Supplementary material for: Computational characterization of Iron metabolism in the Tsetse disease vector, Glossina morsitans: IRE stem-loops
Source: BMC Genomics. 2016 Aug 8;17:561. doi: 10.1186/s12864-016-2932-7 (PMC4977773; doi:10.1186/s12864-016-2932-7)
Supplement: Additional file 2: Figure S1. — Pattern distribution of the identified IRE stem loop structures. Bar graph presentation of the identified IRE-regulated genes, having canonical and non-canonical IREs. Figure S2. GO-category assignments of IRE-regulated genes in Glossina. A bar graph presenting the number of putative IRE-regulated genes associated with each GO category. Figure S3. Sub-cellular localization assignments of IRE-regulated genes in Glossina. A bar graph, presenting the number of putative IRE-regulated genes and their associated subcellular localizations. (DOCX 357 kb) [file 12864_2016_2932_MOESM2_ESM.docx]

**Figure S1**: Pattern distribution of the identified IRE stem loop structures. From the putative IRE structures identified, the majorities resemble non-canonical forms, while all identified structures are similarly distributed between the 5’- and 3’-UTRs. Canonical refers to a stem loop with a terminal loop sequence CAGUG(N) and a 5’ unpaird C(C8) in the stem. Non-canonical stem loops refer to IRE structures derived from SELEX experiments indicating larger terminal loop and more complex patterns of unpaired nucleotides in the C8 region. Here 5UTR-Can = 5UTR-canonical; 3UTR-nonCan = 3’UTR-noncanonical.

**Figure S2**: GO-category assignments of IRE-regulated genes in Glossina. The identified IRE-regulated genes were assigned to six major categories with Transcription and translation (P-value = 5.82e^-5^) as well as metabolism (P-value = 1.39e^-3^) being the over-represented functions using BiNGO as implemented in Cytoscape. Other functional classes such as “Cell envelop” as well as “Transport & Binding” have also been identified, which are of great importance when addressing iron trafficking in tsetse fly.

**Fig. S3**: Sub-cellular localization assignments of IRE-regulated genes in Glossina. The identified IRE-regulated genes were assessed for their sub-cellular localizations to further assist in understanding their functions. Accordingly, the majority of the IRE-regulated genes are cytosolic and nuclear-localized. This is inline with the over-abundance of “Transcription & Translation” observed in the Go-analysis as it is well established that nucleus is the site for such functions.
