# Supplementary material for: Computational characterization of Iron metabolism in the Tsetse disease vector, Glossina morsitans: IRE stem-loops
Source: BMC Genomics. 2016 Aug 8;17:561. doi: 10.1186/s12864-016-2932-7 (PMC4977773; doi:10.1186/s12864-016-2932-7)
Supplement: Additional file 3: Table S3. — Motifs identified in the 5′-UTRs of IRE-regulated genes in Glossina, using MEME and UTRScan. Table S3 presents a list of motifs identified in the 5′UTRs of putative IRE-regulated genes. Table S4. Motifs identified in the 3′-UTRs of IRE-regulated genes in Glossina, using MEME and UTRScan. A summary of the regulatory motifs identified in the 3′UTRs of IRE-regulated genes. (DOCX 2123 kb) [file 12864_2016_2932_MOESM3_ESM.docx]

**Table. S3**: Motifs identified in the 5’-UTRs of IRE-regulated genes in Glossina, using MEME and UTRScan.

**Table. S4**: Motifs identified in the 3’-UTRs of IRE-regulated genes in Glossina, using MEME and UTRScan.
